# Improving justification of medical exposures using ionising radiation: considerations and approaches from the European Society of Radiology

**DOI:** 10.1186/s13244-020-00940-0

**Published:** 2021-01-06

**Authors:** Steve Ebdon-Jackson, Guy Frija, European Society of Radiology

**Affiliations:** 1 Medical Exposure Regulatory Infrastructure Team, CRCE, Public Health England, Chilton, Didcot, UK; 2 Descartes University Paris, Paris, France; 3 European Society of Radiology, Vienna, Austria

**Keywords:** Justification, Ionising radiation, Appropriateness

## Abstract

This discussion paper has been produced within the context of the European Society of Radiology EuroSafe Imaging initiative and considers primarily the issues and challenges associated with justification of medical exposures using ionising radiation for individual patient diagnostic imaging procedures. It addresses both regulatory requirements and practical considerations and discusses approaches that are intended to improve justification.

## Key points

- Justification, an important principle in radiation protection, requires that the benefits of the use of radiation outweigh the associated risks and hazards.
- Appropriate justification is important for ensuring efficient use of resources when using ultrasound and magnetic resonance imaging.
- Before the exposure takes place, a clear understanding of responsibilities for justification and justification itself are needed.
- Imaging referral guidelines remain the most effective tool in ensuring appropriate justification.

## Introduction

Justification is the first, and for many the most important of two fundamental principles in radiation protection that apply to the medical use of ionising radiation (the other being optimisation). It requires that the benefits of the use of radiation outweigh the associated detriments (hazards and risks).

The need to focus on justification was identified as a priority at the time of the publication of the European Council Directive 97/43/Euratom [1], which addressed medical exposures. Since then, the use of medical imaging in modern healthcare has continued to grow. It has been estimated that in Europe during the period from 2007 to 2010, more than 660 million diagnostic imaging procedures (including dental procedures) were performed each year [2]. The most significant growth is in the use of computed tomography (CT) and this has been estimated at approximately 10% per year. CT is thought to account for approximately 70% of patient dose in both the USA [3] and in Europe [4]. While dose per examination for many examinations has been reduced through technological improvements and dose awareness among professionals, its increased availability and acceptance as a mainstream imaging modality has meant that individual patients will routinely undergo CT as part of a healthcare episode and, as a consequence, in many cases doses received during medical exposures have increased. The stochastic risks for the individuals associated with diagnostic imaging are based on models and are generally estimated as being very small but are not always seen as insignificant, especially for children and particularly for conditions where multiple imaging series are required, although there has been no clinical proof of any harm. In addition, inappropriate use of imaging can divert resources from others in greater need.

While the value of appropriate diagnostic imaging in healthcare is recognised and accepted, concern remains around the number of examinations which are performed that are not appropriate and provide limited or no clinical benefit. This has been known for some time. In 1990, the UK’s Royal College of Radiologists and National Radiological Protection Board estimated that 20% of procedures were likely to be clinically unhelpful [5]. Subsequent estimates range from 20 to 50%, depending on the healthcare system, modality considered and the availability of current imaging referral guidelines [6, 7]. In particular, higher levels of inappropriate imaging are evident where the practice of self-referral is prevalent [8, 9]. As a result, appropriate justification has become a key component of a number of major international initiatives, in particular the International Atomic Energy Agency (IAEA) “triple A” approach addressing Awareness, Appropriateness and Audit [10] and its joint initiative with the World Health Organisation (WHO)—the IAEA/WHO “Bonn Call for Action” of 2012 [11]. These initiatives are relevant across Europe and consistent with the requirements of the Euratom Basic Safety Standards Directive (BSSD) [12] where this applies. Appropriate justification of medical imaging procedures is also consistent with basic patient safety and accepted ethical approaches in medicine, including the World Medical Association Declaration of Geneva (2017) [13].

## Scope

This paper addresses justification of individual diagnostic imaging exposures. Its aims are to identify important aspects relating to justification, highlight key requirements and consider approaches and developments which are intended to improve justification of these exposures. Some of the issues raised have applicability to interventional radiology and nuclear medicine imaging and imaging using non-ionising radiation but these are not the primary focus of this paper. Justification of therapeutic medical exposures and types of practice are not considered.

The majority of diagnostic imaging is utilised in the first presentations of adult patients with associated symptoms, or as part of their subsequent care. Presentations by paediatric patients can be a small but important proportion of some radiology services. For paediatric patients, increased radiation sensitivity and greater life expectancy means greater scrutiny should be applied to justification (and optimisation) of imaging. Imaging of asymptomatic individuals whether participating in screening programmes or being exposed as part of individual health assessment (IHA), poses other challenges as these individuals have no obvious clinical condition requiring attention. Those in screening programmes will have been invited to participate and belong to a well-defined cohort. In these cases, individual justification will be relatively simple and will focus on contra-indications such as availability of alternative recent imaging which may make unnecessary further exposures as part of the programme. For those presenting for IHA, probability of a condition is often substituted for symptoms when assessing appropriate justification, but there is little evidence to support the validity of such an approach. There is added complexity when considering sub-types of IHA. Testing individuals for genetic conditions or where there is a national prevalence of some cancers may seem justified on medical grounds. Sports performance monitoring and employer requested exposures relate to fitness to undertake activities and are likely to be undertaken for economic benefit. At present, there are limited publications on the use of imaging in these circumstances [14] but it presents a significant issue for many healthcare economies.

The use of imaging using non-ionising radiation should be considered where appropriate for all patients, but it is particularly important for paediatric patients because of the factors indicated above. Appropriate justification is still important to ensure efficient use of resources when using ultrasound and magnetic resonance imaging (MRI), but issues relating to patient safety are significantly fewer for most imaging when the radiation effects alone are considered – while use of non-ionising radiation has some associated risk, it is considered trivial for most diagnostic applications. At present, the use of imaging using non-ionising radiation for asymptomatic individuals in screening programmes is limited to ultrasound (e.g. thyroid screening following radiological incidents) while the use of non-ionising radiation is not widespread for IHA, although this may grow.

## European and international standards and regulatory requirements

Within healthcare, all clinical services are regulated to some degree. Emphasis changes depending on the discipline, but in general regulation is of the facility, training requirements for staff etc. but not of individual professional actions of staff providing the service. Because of regulatory requirements relating to radiation protection in all sectors, the use of ionising radiation in medicine is further regulated, including the justification of individual medical exposures. This is direct regulation of clinical practice.

For most of Europe, regulatory requirements relating to radiation protection are provided by the 2013 Euratom BSSD. This directive includes medical exposure for the first time, such exposures having been addressed previously in a separate Directive - 97/43/Euratom, where justification was already addressed. The BSDD is goal-setting and in its direction to Member States, it allows some flexibility for national regulations to reflect local healthcare provision, systems and culture. In contrast, however, the competent authorities charged with regulatory oversight are required to create clear and unambiguous regulations which state where responsibilities rest for all the elements of the justification process.

In Europe, a survey undertaken in 2020 by EuroSafe Imaging through the Heads of the European Radiation protection Competent Authorities (HERCA) channel (see Additional file 1) of 19 European Union Member States revealed that 22% of competent authorities responsible for radiation protection for medical exposures are part of the Ministry of Health or other Ministry, 38% are linked to the Health Ministry (e.g., as an Agency of the Health Ministry) while 38% are independent of the national Health Ministry. While there are advantages of independence, clear separation can lead to a lack of appreciation of each organisation’s priorities, which are not always consistent. For example, a Health Ministry may promote health self-awareness and support early diagnosis through rapid referral for imaging, while not fully understanding the regulatory requirements for justification by an imaging specialist, rather than a referring family doctor who is not appropriately trained.

It is possible to have consistent policies in both cases, although separation requires regular liaison and policy alignment. Excellent public awareness campaigns regarding the unnecessary use of imaging using ionising radiation have been launched in Belgium, where the radiation protection competent authority (FANC) is a separate entity, and Luxembourg, where the equivalent authority is integrated within the Health Ministry.

Justification of individual exposures is the first article of the chapter on medical exposures (Article 55) and it includes familiar concepts and requirements, including the need for benefit to outweigh detriment and that exposures are justified in advance. This second requirement provides challenges in busy imaging departments. Other articles address justification, most notably Article 57 which outlines responsibilities and states that the referrer and the practitioner are involved in the justification process, as specified by Member States. In total, justification is addressed in three articles within the BSSD’s chapter on medical exposures, thus complicating any operational approach to the justification concept.

The International Basic Safety Standards includes similar requirements, which are not legally binding in the way that the Euratom BSSD is on EU Member States. In particular, it is less specific when addressing the responsibilities for justification, requiring a consultative approach involving the radiological medical practitioner and the referring medical practitioner as appropriate.

## The justification process—roles and responsibilities

Before exploring the practical implications and solutions for justification, it is useful to consider in some detail the requirements and associated responsibilities relating to the justification process, as stated in the BSSD.

It should be noted that the Directive refers to the justification process rather than justification alone and this is an important distinction. The justification process includes a number of sequential and parallel activities beginning with the initial presentation of the patient and ending with the authorisation for an exposure to take place. It is helpful to consider these as separate elements of a process and to assign clear functions to each of these steps. Turning these functions into roles within a clear regulatory framework can then be transparent and responsibilities can be assigned to the person best placed to carry them out, based on their training and their role in the patient’s healthcare pathway.

A well-constructed regulatory approach may allow for local arrangements to reflect needs and practice for different scenarios or healthcare settings. By doing so, there is greater likelihood of compliance. It is important, however, that all local arrangements are consistent with the regulatory framework.

It is also possible for local arrangements to maintain some flexibility regarding those who are responsible for justification for a specific investigation. Processes should allow for responsibility for justification to be transferred from one professional to another where it is clear that the first professional does not have the competence to make an appropriate decision. In such cases, this should be documented.

The EuroSafe Imaging/HERCA survey referred to above has provided additional information regarding roles and responsibilities for justification in imaging. Further detail regarding the survey is summarised in the Additional file 1. This survey illustrates the views of competent authorities on regulatory requirements, following the transposition of the EURATOM BSSD. As such it reflects current legal requirements and approaches.

### Definition of the practitioner in radiology

Article 2 of the BSSD defines both the referrer and practitioner as well as clinical responsibility. The referrer is defined as a health professional who is entitled to refer individuals for medical radiological procedures to a practitioner. The practitioner is defined as a healthcare professional who is entitled to take clinical responsibility for an individual medical exposure (in accordance with national requirements) and the definition of clinical responsibility refers directly to the responsibility of a practitioner for individual medical exposures. Although Article 57 allows for the involvement (as specified by the Member State) of the referrer and the practitioner in the justification process, it also reaffirms that the clinical responsibility for the medical exposure rests with the practitioner. It is reasonable to assume therefore that the primary responsibility for justification rests with the practitioner. It would be interesting to see how this concept of responsibility is implemented practically across Europe.

In the USA, where the BSSD does not apply, the referring clinician is considered to be responsible for justification as part of the referral and ordering process. For more complex examinations, the referring clinician will normally consult with the radiologist before a request/order is made. This is particularly common for paediatric patients, but responsibility remains with the referrer. In such cases, adoption of CDS systems enhances the referrers knowledge of appropriate medical exposures and does not challenge existing legal responsibility.

The BSSD definition of practitioner allows for a range of healthcare professionals to take on the role. The EuroSafe Imaging/HERCA survey has demonstrated how this is approached in practice. The survey indicated that apart in UK and Ireland the Member States defined in national legislation the practitioner as a medical doctor (58% indicated this was a radiologist). The UK and Ireland responses indicated that other healthcare professionals such as radiographers and nurses could be entitled to act as practitioners, and allowed responsibility to be assigned to non-medically qualified staff as long as this was restricted to certain staff groups (e.g., radiographers and nurses) and that the scope of this activity was well defined and supported by adequate training. Further comments explained that in some Member States a range of medical practitioners were allowed to be practitioners for specified fields and that in one case the undertaking itself could be considered as the practitioner, with the activities undertaken by authorised health professionals.

### Responsibility and delegation of tasks

While the requirement to transpose and implement European Directives, and hence the BSSD, is common for all European Union Member States, the way they do so is not and reflects existing national legislative systems and regulations. One feature of many European legal approaches is that responsibilities as laid out in regulations cannot be delegated or transferred, but an associated task can be, as long as the person to whom the task is delegated is competent.

The EuroSafe Imaging/HERCA survey indicated that the legislation of 58% of respondents allowed medical tasks in general to be delegated to non-medically qualified staff, while 42% did not. With regards to justification of radiology examinations, 37% indicated this was allowed (for 63% of respondents this was not permitted) as long as responsibility remained with a medical practitioner. Where the task of justifying was permitted, this needed to be documented, restricted to certain staff groups or individuals within these groups (e.g. radiographers and nurses) and the competence of these individuals must be assessed. In many cases, delegation of tasks only applied to the justification of simple procedures and did not include CT.

The EuroSafe Imaging/HERCA survey demonstrates that many Member States have chosen to exploit the flexibility of the Euratom BSSD. Radiologists however remain central in establishing and agreeing policies and procedures relating to justification, including assigning of responsibilities and delegation privileges to other medically and non-medically qualified staff for radiology medical exposures.

### Potential impact of CDS on roles and responsibilities

The introduction of integrated requests and CDS systems have the potential to challenge the existing and accepted allocated responsibilities for justification throughout Europe. CDS systems provide a direct link between the referring clinician and those administering and performing imaging and in many cases the radiologist’s direct input prior to the examination taking place could be by-passed for many examinations. In such cases, the responsibilities of all those involved in the pathway from original patient presentation to justification of a medical exposure should be reviewed and clarified.

In Europe, Article 18 of the Euratom BSSD and national transposing national regulations require the practitioner to have adequate education, information and theoretical and practical knowledge, specifically relating to radiation protection. While some referring clinicians may have achieved this in a limited field, many will not have done so and to become a practitioner, such clinicians, including most family doctors, will need to have undertaken additional training in radiation protection. Most general referrers will be reluctant to undertake such training and while good CDS systems may increase the percentage of appropriate requests, they will not address this need for additional training for the practitioner, as required by the BSSD and national legislation.

To satisfy European legal requirements, it may be necessary for the responsibility for justification for radiology procedures to remain with the radiologist or other radiology professional, even when CDS systems are in place. This might be achieved by a named radiologist, such as the radiology department’s Clinical Director, assessing the applicability of the CDS system to local practice and formally accepting responsibility for all examinations performed in accordance with the recommendations of the CDS system. Where requests and the CDS system’s recommendations are not consistent, the functionality of the system should identify these, and initiate processes that allow for appropriate review and justification by a radiologist, prior to the examination taking place. The option to review any individual request would remain, either when the request is first received through the integrated CDS system or other mechanism, or immediately prior to the examination taking place. In such cases, staff other than the Clinical Director could receive, review and amend if necessary, the original request, following consultation with the referring clinician, and take on the role of practitioner for the procedure now agreed. In such cases, this process should be documented in accordance with local polies and procedures.

Fully integrated CDS systems are expected also to facilitate retrospective review and audit of requests, thus improving the requesting and justification process and informing the development and amendment of CDS recommendations as practice changes and is agreed.

### Reducing conflict between regulation and practice

In most cases, roles and responsibilities in justification can be made clear and acceptable to all clinical professionals involved with the healthcare pathway of any individual patient. Regulatory requirements serve to formalise good practice and should not impact negatively on service provision as long as it is robust and of high quality. In this respect, the regulator and the healthcare professional should have common aims. By adopting an approach of clarity and transparency regarding roles and responsibilities, radiology services can demonstrate legal compliance to the satisfaction of regulators. Involvement of radiologists is essential when establishing a service’s policies and procedures for imaging, including appropriate justification, as well as undertaking justification of individual examinations where their input is required directly.

## Challenges

While the regulatory approach and requirements described above need to be understood and complied with, there are other issues and competing priorities which need to be recognised and addressed.

### Patient expectations

The regulatory structure for radiation protection was originally designed for the nuclear industry and industrial uses of ionising radiation. Extension into medical exposures was inevitable as it became clear that the largest radiation doses received by the population from man-made sources were from medical uses, but extension is not without problems. Most non-medical uses of ionising radiation rely on processes to assess justification of types or classes of practice, whereas medical exposure requirements focus on justification of individual exposures and the direct interaction with the patient.

In recent years, with the advent of greater information availability and growing consumer confidence and power, the delivery of healthcare has had to take into consideration the patient’s wishes to a far greater degree than ever before and the traditional paternal approach in medicine is no longer accepted by the public, patients or the professions involved. Radiology services are not immune to this.

The effects of radiation are not well understood by the public and perception of risk varies enormously depending on factors such as whether the radiation is from a natural source or man-made, and whether the individual can choose the exposure or if it is imposed in some way. The public generally accepts that imaging is a part of healthcare and any concerns about radiation exposure are usually overridden by a desire for imaging to be performed (the common exception being imaging of children). If patients’ expectations are to be managed, then communication about the value of imaging is essential and family doctors, medical specialists, other appropriate healthcare professionals, as well as radiologists must be willing to advise patients when imaging is of value and when it is not appropriate.

The regulatory requirements around justification support this, but it must be acknowledged that saying “no” is not easy for a healthcare professional when in direct contact with a concerned patient, as it can lead to a breakdown of trust on the part of the patient. It also takes time. Public health initiatives are extremely useful for informing the public that radiological imaging is not without risks and should not be demanded. The excellent examples of these in Belgium and Luxembourg referred to previously and an information campaign launched in 2020 by HERCA are all aimed at supporting family doctors [15].

### Allocation of resources

Perhaps the greatest challenge facing radiology services and staff is the sheer volume of work, and the requirement to maximise the impact of key staff and in some cases the limited number of staff available. Imaging is a key part of most healthcare pathways and the positive impact of imaging on patient management means that radiology services are becoming a victim of their own success. Yet at the same time, these services are considered expensive because of high equipment costs and funding is seldom sufficient to expand services so that they can address increased demand for greater numbers and more complex imaging.

In these circumstances it is essential that radiology departments should evaluate their services, ensuring that radiologists focus their attention regarding justification where their input is most efficient and effective i.e. on the application of new or complex techniques, rather than existing and well-established ones. This applies particularly if such imaging has been incorporated into evidence-based protocols and clinical pathways, agreed by a multi-disciplinary medical team and facilitated by computerised decision support (CDS) systems. Nevertheless, a clear understanding of responsibilities for justification is still needed and justification is required before the exposure takes place - a specific requirement of the BSSD and therefore of national regulations.

### Safety culture

In the past five years, the importance of safety culture in radiological services has come to the fore. This is consistent with the way all healthcare is delivered and the multi-disciplinary nature of radiology departments makes them prime candidates for adoption of the safety culture ethos. Key components of safety culture include openness and transparency, including the creation of an environment where all professional staff feel they can draw attention to practices they feel may be inappropriate and detrimental to patient safety.

A healthy safety culture must be promoted and supported by management and should be evident in public and private practice imaging departments, allowing staff to openly and professionally question the actions of others. In practice, safety culture in radiology is likely to have its greatest impact when considering practices around justification rather than individual cases. Mechanisms should be put in place to encourage staff to be able challenge practice which does not seem in the best interest of patients, staff or the department as a whole. Similarly, mechanisms must be introduced to enable conflict review and resolution. Where these are in place, all staff groups are more likely to embrace a safety culture mentality.

While safety culture is important, it should not be used to bypass existing responsibilities laid out in policies and procedures. Where responsibility for justification of an individual exposure is clear and explicit, e.g. it lies with the radiologist, then his or her knowledge, competence and skills, should be recognised and respected. Challenges to appropriateness in such cases should be made through the agreed mechanisms and should be addressed by the practitioner involved.

### Healthcare pathways

The framework provided by the fundamental radiation principles of justification and optimisation is clearly applicable to medical exposures but does not reflect completely the way medical exposures in healthcare are often delivered in practice. Within the BSSD, the principle of justification is applied to types or classes of practice (not addressed here) and individual exposures. In healthcare however, imaging is often applied as part of a pre-defined healthcare pathway including initial assessment and periodic follow up to assess either disease progression or efficacy of treatment. Radiological imaging is often used in both phases and therefore involves multiple imaging for a health episode but over a prolonged period of time. In these circumstances, an initial imaging procedure may be justified on the understanding that further imaging will take place and yet justification of subsequent imaging procedures is usually considered as a separate exercise. Until the initial imaging takes place, however, it is impossible to be sure that further imaging will be needed and take place. There is clearly a disconnect. For example, lymph node assessment by CT may be justified in cancer staging, followed by a further PET-CT study to help determine patient management. These studies could be justified separately, and in some cases by different imaging specialists within different imaging services, in accordance with the concepts of individual justification and resource availability and management.

It may be more realistic to justify a series of exposures on the basis of an expected care pathway and then reassess justification of some or all of subsequent procedures within the care pathway as the patient’s condition develops and care is subsequently modified. At the same time, different approaches can be considered for optimisation of subsequent imaging.

It is important therefore that radiologists are involved when care pathways involving imaging are being devised and established. Doing so may remove possible conflicts between radiologists and referring physicians as well as help resource planning within radiology departments. While justification of a series of diagnostic imaging at the outset may not fit in well with a regulatory approach, it may make more sense in a medical setting and may have implications for the way justification is carried out. Justification of each imaging exposure will still be required, but if such imaging is part of an agreed protocol then it might not need a radiologist to do it at all stages.

There will however be circumstances where it becomes apparent that some imaging procedures within a care pathway do not provide useful information for a specific patient and the imaging investigation should not be performed. Examples include patients whose clinical condition is resolved before the end of the pathway, either by cure or by a failure to respond as expected.

In such cases, actively reviewing or de-justifying exposures within a pathway may become an important activity, saving radiation exposure, reducing interventions for individual patients and freeing up resources for others. This would require radiologist input or agreement and would mark a significant difference in the way justification processes are seen today. In most cases, changes to initial justification of individual exposures within a pre-agreed series would require medical knowledge and could be performed and documented when considering individual imaging requests or preferably as part of multi-disciplinary meetings attended by a range of medical specialists involved in the care of a specific patient. The role of radiologists in such meetings is well established in most of Europe and even mandatory in Cancer care in some countries (in France for example).

## Practical tools, professional initiatives and inspection

### Imaging referral guidelines

Imaging referral guidelines have been in place for many years in Europe. The intention of the guidelines is to help the referrer, rather than the radiologist, and to provide an indication of practice. In the USA, appropriateness criteria have been developed, which might be considered as standards against which a proposed radiological investigation can be assessed. Both approaches are valid and tend to use the same evidence base in their generation but reflect different approaches to responsibility for justification. It is worth noting that in the European BSSD, requirements for referral guidelines are intended for referrers and included in the article addressing procedures and not that for justification.

In the UK, and subsequently in France, referral guidelines were originally intended to help the referrer make the best use of the imaging department [16, 17]. This imperative remains but the emphasis has subtly changed in the UK and in other countries where referral guidelines have been produced. Notwithstanding, the value of imaging referral guidelines as part of the justification process has been shown in a number of studies [18, 19] and the importance of availability and use has been investigated by the European Commission and the ESR [20, 21]. In an ESR internal survey (2011), it was shown that although guidelines were existing and accessible, they were not widely used in the following states: Belgium, France, Germany, Hungary, Ireland, Italy, The Netherlands, Spain and Switzerland.

For imaging referral guidelines to be effective, they need to be up to date, consistent with local clinical and radiological practice, available and easily accessible at the point of care and used. Consistent use of guidelines requires their integration into normal workflow without which they will become an afterthought. Numerous publications have demonstrated this and initiatives making available stand-alone guidelines have to be repeated and reinforced if use is to be maintained. Other studies suggest poor utilisation or inconsistency between guidelines and practice could be explained because the published guidelines did not meet these requirements. A study in Sweden in 2009 [22] showed large discrepancies between CT referrals and performed examinations and available guidelines (the EC produced RP No 118) [23], but this might have been expected as these guidelines reflected practice from the mid-1990s and pre-dated the availability and capability of multi-detector CT.

The perceived relevance of guidelines is also influenced by their intrinsic limitations. Guidelines are population based and may not be considered appropriate for individual patient’s presentations. Reliance on consensus rather than robust published evidence and lack of transparency reinforces different views and approaches. Failure by the radiology community to include the views of other specialties when generating guidelines has often resulted in inconsistencies between physician developed protocols and radiologist produced imaging guidelines. Early pre-authorisation systems developed in the USA resulted in decreased numbers of procedures undertaken, rather than more appropriate imaging taking place, and this was viewed as a major drawback. Nevertheless, experience in Israel of use of the American College of Radiologists’ appropriateness criteria and RCR referral guidelines have shown an efficiency impact on the justification process [24].

Finally, acceptance of imaging guidelines will be affected by the importance given to them by health policy makers and regional or national Ministries of Health. The necessity for referral guidelines, as required by the Euratom BSSD and resulting national regulations, may not be fully appreciated by those responsible for health provision and provision and use of imaging guidelines may not be considered a priority.

### Computerised decision support (CDS) systems

To address some of these issues, in recent years more comprehensive CDS systems have been developed, integrating imaging referral guidelines into electronic requesting systems. The greatest experience of the benefit of these comes from the USA where the use of CDS was initially expected to become mandatory by 2020, and where a number of studies have shown an increase in the appropriate use of imaging within the context of an overall reduction of the number of examinations performed. In Europe, the ESR’s iGuide shows considerable promise and it is generally accepted that this approach will be productive. Although evidence demonstrating this is limited currently, the experience in Croatia is encouraging [25].

These early successes all highlight the need for a supportive environment, and this support is essential from health policy makers at national and local level, both for initial introduction and on-going change management. Education of referring physicians and integration into healthcare pathways and workflow are also required. Shared experience of early adopters will be essential to promote more general uptake of integrated CDS across all radiology services and if this experience relates to European models of healthcare delivery then it is likely to be quicker. The introduction may follow the same pathway as the introduction and adoption of picture archiving and communication systems (PACS) in the 1990s, and of course PACS is now ubiquitous and its value is accepted. A key factor for CDS, as with PACS, will be availability and ease of use at the point of care by referring physicians.

### Education and training

Education and training of healthcare professionals provides the knowledge and competence required to undertake their activities and this will vary for those involved in the justification process for radiological imaging. All staff involved in radiology and nuclear medicine imaging should have sufficient training to understand their roles and responsibilities, if any, as outlined in the national regulation derived from the BSSD.

Those specialising in radiology and nuclear medicine will be expected to keep up to date with developments in radiation protection as well as advances in clinical and therapeutical knowledge and the application of new techniques in their field. In addition, consideration of the benefits of non-ionising radiation imaging modalities must be regularly updated and shared with other non-imaging physicians. Medically qualified imaging specialists will be adequately trained to be referrers and practitioners in their own specialty.

Specialists in other fields (e.g., orthopaedists, interventional cardiologists etc.) may have sufficient medical and radiation protection training to undertake justification for a limited scope of practice.

Patient assessment is an essential part of all doctors’ activities so they should not require additional training to be referrers. Most family doctors and many hospital doctors, however, are unlikely to have up to date knowledge of imaging or radiation protection and they should not take on the role and responsibilities of the practitioner, without undergoing considerable further training, including detailed radiation protection training. Nevertheless, some knowledge is helpful for all clinical staff who act as referrers for imaging, to support their interaction with patients. For example, a family doctor should be able to provide limited information about the preparation for different imaging procedures and the relative risks of each and be able to put this into perspective when considering and discussing the patient’s potential condition. Guidance is available to support this.

### Audit

Evidence of the value of CDS and its impact on justification can be produced by clinical audit which is itself a requirement of the BSSD. Guidance on clinical audit has been produced by the European Commission [26] and will be subject to further study by the Commission. The ESR has developed a practical audit tool - Esperanto [27] - and there is a long history of clinical audit in radiology in both UK and Finland [28–30]. Up to now, audits of appropriate justification have required manual processes which are time consuming. Comprehensive CDS systems offer the option of real-time review of individual cases, patterns of referrals and subsequent justification of examinations, and may influence requesting practice by offering feedback to referrers. This should make review of practice much easier and quicker, including the resetting of standards and inform subsequent data collection exercises for imaging referral guideline generation.

Clearly, the generation, availability, use and audit of the role of imaging referral guidelines in justification are inextricably linked. CDS could change significantly the willingness to undertake audit, the frequency that this is done and consequently raise the profile importance of awareness and appropriateness with referrers. Recently, the European commission launched a tender on audit of CT justification [31], showing the actuality of this topic.

### Inspection

Inspection of clinical practice by a regulatory body is unusual in medicine but the BSSD requires this for medical exposures. In practice the focus is on processes rather than individual actions and this applies to the justification process.

Inspectors will assess compliance with local procedures and regulatory requirements for justification. Specific elements of the process will include assessing whether an institution’s policies and procedures are for purpose, identification of the referrer and practitioner, the competence of individuals to act in these capacities (i.e., education and training records), verification that clinical data was provided to support the justification of a specific imaging procedure and that the procedure was justified prior to the exposure taking place.

In some cases, the education and training of the inspector may be sufficient to discuss or challenge justification of procedures for individual patients. This remains unusual in Europe.

In 2015, as part of its activities to demonstrate compliance with regulations, HERCA’s Working Group on Medical Applications (HERCA WGMA) instigated an inspector workshop on justification in radiology and in 2016/2017 organised a coordinated European Action Week on the inspection of justification involving 148 inspections in 17 countries [32]. The results showed significant variation across Europe and a need for increased awareness of the importance of justification.

While inspection is a useful tool, aimed at demonstrating compliance with regulatory requirements rather than enforcement, it has limited impact on day-to-day practice unless the importance of justification is recognised by professionals and professional bodies. HERCA WGMA is continuing to work with professional bodies and will launch other initiatives in the future.

## Justification as a quality indicator

Appropriate use of imaging has benefits both for individual patients and for the efficient use of any radiology service, whether within a radiology department or across the wider institution where imaging takes place at multiple locations. Analysing justification of exposures therefore provides key information regarding quality of a service’s performance and of those that work within such services.

This should be accepted within the healthcare institution but could be considered also as a key performance indicator in any external accreditation programme or quality assessment of a radiology department.

The EuroSafe Imaging Stars initiative is intended to identify and recognise imaging facilities with high standards relating to radiation protection. In addition, it provides opportunities for participating institutions to learn from others and improve from the experience of others. It features five self-evaluation criteria for justification including evaluation of requests for cross-sectional imaging, identification of the referrer, the availability of referral guidelines, the use of CDS and a justification policy for pregnant patients. At present, the criteria identified are the basis for self-evaluation and evidence is only required for the availability of referral guidelines within the institution and the use of CDS. Future development of the EuroSafe Imaging Stars initiative might consider extending this and including justification as part of any external evaluation carried out to validate the institution’s own assessment.

## Discussion and conclusions

Appropriate justification remains one of the most important challenges for radiological services and staff, whether considered from the perspective of patient safety and radiation protection or the efficient use of radiological resources, be they equipment and facilities or workforce. While there is evidence of improvement in European countries, it is essential that complacency does not reverse this trend. Justification is central to the goal of ensuring patient safety and appropriate care while simultaneously achieving better use of imaging facilities, better use of radiologists’ and radiographers’ knowledge and skills within these facilities and better integration of imaging into the wider provision of healthcare at local and national levels.

Imaging referral guidelines remain the most effective tool in ensuring appropriate justification. The value of referral guidelines has been established, but only if they are used routinely. The European Commission and others recognise this and greater emphasis is being given to availability and use rather than production. Simple, one-click computer-based systems with integrated referral guidelines, designed to address requesting and selection of imaging procedures, have the potential to improve further the impact of referral guidelines. Adoption is likely to be slow at first, but there is sufficient evidence to support introduction on a wider scale. Like the introduction of PACS in previous decades, audit and research relating to the impact of introduction will accelerate uptake. Commitment to continued support for generating up to date evidence-based guidelines and mechanisms to ensure these guidelines are readily available will be essential. The continued development of ESR’s iGuide is central to this, as is its evaluation through audit, without which the case for adoption will be weak.

Comprehensive integrated CDS and requesting systems may be the way forward, but the impact on professional roles and responsibilities regarding justification must be discussed and agreed in a meaningful manner. These may differ for different types of imaging, taking into account modality and complexity.

When discussing roles and responsibilities, it is impossible to ignore the need for regulatory compliance. The flexibility of the BSSD allows Member States to consider how responsibilities can be better allocated for the justification process, reflecting the way justification of imaging is undertaken at a local level. While this flexibility can be exploited at local level, it should be recognised that any flexible approach should still be formalised within local procedures so that responsibilities associated with the justification process are clear and well understood.

Patients’ expectations can be influenced by public health campaigns and coordinated approaches with family doctors. This will require active and constructive dialogue with organisations including health authorities, competent authorities for radiation protection and professional bodies representing those responsible for providing primary, secondary and tertiary healthcare.

Similar dialogue will be essential if diagnostic imaging is to be used appropriately in healthcare pathways. Justification of series of exposures for specific patient protocols or care pathways is likely to become important and may change approaches to justification, which currently focus on individual exposures.

Audit and inspection are both designed to ensure good practice but are separate processes and one does not replace the other. The importance of audit is likely to grow as it provides a continuing platform for assessment and improvement. Justification is an ideal audit topic and the introduction of CDS may encourage more comprehensive and frequent assessment.

## Supplementary information

**Additional file 1.** Results of a EuroSafe Imaging survey, carried out via the Heads of the European Radiation protection Competent Authorities (HERCA).

## Data Availability

All data and materials are published in this article.
