# Supplementary material for: Improving justification of medical exposures using ionising radiation: considerations and approaches from the European Society of Radiology
Source: Insights Imaging. 2021 Jan 6;12:2. doi: 10.1186/s13244-020-00940-0 (PMC7788115; doi:10.1186/s13244-020-00940-0)
Supplement: Supplementary file 1 — Additional file 1. Results of a EuroSafe Imaging survey, carried out via the Heads of the European Radiation protection Competent Authorities (HERCA). [file 13244_2020_940_MOESM1_ESM.docx]

**Improving justification of medical exposures using ionising radiation –**

**considerations and approaches from the European Society of Radiology**

**Electronic Supplementary Material**

| **Please specify how 'practitioner' is defined**  **in your national legislation** | **Responses** |
| --- | --- |
| Medical doctor | 68.42% |
| Radiologist | 57.89% |
| Radiographer | 10.53% |
| Nurse | 5.26% |
| Non specified | 0% |
| Other | 63.16% |

| **Question** | **Yes** | **No** | **Don’t know** |
| --- | --- | --- | --- |
| Does your country specify in legislation or in guidance that the practitioner who justifies a radiology exposure has to be a radiologist? | 26.32% | 73.68% | 0% |
| Can the responsibility for justification of radiology  exposures be assigned to non-medically qualified  staff? NB this question may relate to a range of  specifically trained staff including physician  assistants, radiographers etc. | 26.32% | 73.68% | 0% |
| In your country's national law, can a medical task be  delegated to specifically trained, non-medically  qualified staff, with responsibility remaining with a  medically qualified individual? | 57.89% | 42.11% | 0% |
| Consequently, can the task of justification of  radiology exposures be delegated to non-medically  qualified staff, with responsibility remaining with a  medically qualified practitioner (e.g. radiologist)? | 36.84% | 63.16% | 0% |

| **Is the competent authority for radiation protection for medical exposures…** | **Responses** |
| --- | --- |
| ...part of the Health Ministry | 22.22% |
| ...linked to the Health Ministry (e.g. as an agency of the Ministry) | 38.89% |
| ...independent of the Health Ministry | 38.89% |
